# A case report of a mild form of multiple acyl-CoA dehydrogenase deficiency due to compound heterozygous mutations in the *ETFA* gene

**DOI:** 10.1186/s12920-020-0665-6

**Published:** 2020-01-29

**Authors:** Robin Chautard, Cécile Laroche-Raynaud, Anne-Sophie Lia, Pauline Chazelas, Paco Derouault, Franck Sturtz, Yasser Baaj, Alice Veauville-Merllié, Cécile Acquaviva, Frédéric Favreau, Pierre-Antoine Faye

**Affiliations:** 1 CHU de Limoges, Service de Biochimie et Génétique Moléculaire, F-87000 Limoges, France; 2 CHU de Limoges, Service de Pédiatrie, F-87000 Limoges, France; 3 CHU de Limoges, Centre de Compétence des Maladies Héréditaires du Métabolisme, F-87000 Limoges, France; 4 Université de Limoges, Faculté de Médecine, Maintenance Myélinique et Neuropathies Périphériques, F-87000 Limoges, France; 5 CHU de Lyon, HCL, Service de Biochimie et Biologie Moléculaire, Unité Maladies Héréditaires du Métabolisme, F-69677 Bron, France

**Keywords:** MADD, ETFA, Mild form, Hypoglycaemia, Compound heterozygous mutation

## Abstract

**Background:**

Multiple acyl-CoA dehydrogenase deficiency (MADD), previously called glutaric aciduria type II, is a rare congenital metabolic disorder of fatty acids and amino acids oxidation, with recessive autosomal transmission. The prevalence in the general population is estimated to be 9/1,000,000 and the prevalence at birth approximately 1/200,000. The clinical features of this disease are divided into three groups of symptoms linked to a defect in electron transfer flavoprotein (ETF) metabolism. In this case report, we present new pathogenic variations in one of the two ETF protein subunits, called electron transfer flavoprotein alpha (ETFA), in a childhood-stage patient with no antecedent.

**Case presentation:**

A five-year-old child was admitted to the paediatric emergency unit for seizures without fever. He was unconscious due to hypoglycaemia confirmed by laboratory analyses. At birth, he was a eutrophic full-term new-born with a normal APGAR index (score for appearance, pulse, grimace, activity, and respiration). He had one older brother and no parental consanguinity was reported. A slight speech acquisition delay was observed a few months before his admission, but he had no schooling problems. MADD was suspected based on urinary organic acids and plasma acylcarnitine analyses and later confirmed by genetic analysis, which showed previously unreported *ETFA* gene variations, both heterozygous (c.354C > A (p.Asn118Lys) and c.652G > A (p.Val218Met) variations). Treatment was based on avoiding fasting and a slow carbohydrate-rich evening meal associated with L-carnitine supplementation (approximately 100 mg/kg/day) for several weeks. This treatment was maintained and associated with riboflavin supplementation (approximately 150 mg/day). During follow up, the patient exhibited normal development and normal scholastic performance, with no decompensation.

**Conclusion:**

This case report describes new pathogenic variations of the *ETFA* gene. These compound heterozygous mutations induce the production of altered proteins, leading to a mild form of MADD.

## Background

Multiple acyl-CoA dehydrogenase deficiency (MADD; OMIM #231680), previously called glutaric aciduria type II, is a rare congenital metabolic disorder of fatty acids and amino acids oxidation, with recessive autosomal transmission. First recognized in 1976, its prevalence in the general population is approximately 9/1,000,000 and 1/200,000 live births [1].

Mitochondrial fatty acids oxidation and amino acids metabolism are affected in MADD. These disorders are linked to a dysfunction of electron transfer flavoprotein (ETF) [2]. Normally, electrons generated by fatty acids oxidation in the mitochondrial matrix are first transferred to ETF and then electron transfer flavoprotein dehydrogenase (ETFDH = ETF-Ubiquinone-oxidoreductase = ETF-QO). Finally, the electrons are transported to coenzyme Q, located in the inner mitochondrial membrane. These electrons feed oxidative phosphorylation to produce ATP. ETF, which is a heterodimeric protein, is composed of two subunits: alpha (ETFA) and beta (ETFB). EFTA is composed of two domains and ETFB of one. Several mutations in the *ETFA*, *ETFB*, and *ETFDH* genes have already been described and associated with variable clinical presentations of the disease*.*

The clinical features of MADD can be classified into three phenotypes: i) type I, a neonatal form with congenital anomalies, such as facial dysmorphism and renal cysts associated with severe hypoglycaemia, acidosis, hypotonia, and hepatomegaly, with a poor outcome in the first days of life; ii) type II, also present in early neonatal life, without congenital anomalies, for which survival does not exceed a few months; and iii) type III, which has variable symptoms, often much milder than those of the two other types, which begin during childhood or early adulthood. Type III shows an intermittent course, with severe hypoketotic hypoglycaemia and hyperammonaemia, often associated with fatigability and hepatomegaly during decompensation episodes. It is also characterized by an energy deficiency and the accumulation of toxic intermediates. Treatment for this form generally consists of avoiding fasting combined with riboflavin supplementation (Vitamin B2). This treatment is particularly recommended for patients expressing *ETFDH* gene mutations [3]. Here, we report new pathogenic variations of the *ETFA* gene associated with a mild form of MADD.

## Case presentation

A five-year-old child was admitted for seizures without fever. No personal or familial antecedents nor parental consanguinity were reported. He had one older brother and had been a full-term eutrophic baby with a normal vitality index (APGAR) at birth. Normal early psychomotor development had been recorded. Only a slight delay in speech acquisition had been observed a few months before emergency admission, without any hearing or schooling problems.

The circumstances were as follows. After a very active day and no evening meal, the boy was found unconscious and experiencing tonic-clonic seizures the following morning. In the emergency room, his vital signs were normal, with a Glasgow score of 7. However, glycaemia was 0.3 g/L (30 mg/dL, control range (CR) 0.6 to 1 g/l). Despite glucose supplementation (30% intravenous), he exhibited only a slight gain in consciousness and limited improvement. A second hypoglycaemic episode (0.19 g/L, 19 mg/dL) again led to tonic -clonic movements and unconsciousness. Orotracheal intubation was performed, restoring normal oxygen saturation, but was associated with tachycardia of 140 bpm and low blood pressure (83/36 mmHg). However, no abnormality of the heart, digestive track, or skin was observed and no organomegaly was found. Except for the loss of consciousness, the neurological examination was normal. Laboratory tests showed uncompensated metabolic acidosis and hyperammonaemia at 116 μmol/L (CR < 55 μmol/L). Cerebrospinal fluid biological marker, plasma glycaemic cycle marker (glycaemia/insulin/C-peptide), and cortisol/ACTH hormone levels were all normal. However, total and free carnitine plasma levels were markedly low: 5 μmol/L (CR 43 to 65 μmol/L) and 3 μmol/L (CR 30 to 40 μmol/L), respectively.

These findings all suggested decompensation of a metabolic disease, which was thus explored by metabolic profiling of plasma and urine samples at the Clinical Biochemistry Departments of the Limoges and Lyon University Hospitals. The amino acids profile in plasma was largely non-informative. However, the urinary organic acids profile (performed by Gas chromatography – mass spectroscopy) showed high levels of several organic acids, such as dicarboxylic acids and glutaric acid (Table 1). In addition, a peak of suberylglycine was detected, along with ketosis revealed by high levels of acetoacetic acid and 3 hydroxybutyric acid. Hypo-carnitinemia was associated with a pathological acylcarnitine profile (performed by tandem mass spectroscopy), which showed slightly elevated levels of medium-length acylcarnitine chains, particularly octanoyl (0.6 μmol/L, CR <  0.3) and decanoylcarnitine (1.1 μmol/L, CR <  0.5). After L-carnitine supplementation (100 mg/kg/day), the levels of free and total carnitine increased significantly, rising from 3 to 65 μmol/L and 5 to 85 μmol/L, respectively. The acylcarnitine profile also showed elevated levels of short and medium-length chains (butyrylcarnitine (C4): 1.6 μmol/L (CR < 0.6), octanoylcarnitine (C8): 1.0 (CR < 0.3), decanoylcarnitine (C10): 1.0 (CR < 0.5), and tetradecenoylcarnitine (C14:1): 0.3 (CR < 0.2)). Despite the absence of elevated levels of glutarylcarnitine (C5) in the acylcarnitine profiles, these alterations pointed towards MADD, a riboflavin metabolism defect or a medium-chain acyl-coenzyme A dehydrogenase deficiency (MCAD). Based on these findings, we analysed genes involved in multiple acyl-CoA dehydrogenase deficiency (*ETFA* (NM_000126), *ETFB* (NM_001985), and *ETFDH* (NM_004453)) and riboflavin transport and metabolism (*SLC52A1* (NM_017986), *SLC52A2* (NM_024531), *SLC52A3* (NM_033409), *SLC25A32* (NM_030780), *FLAD1* (NM_025207), and *RFK* (NM_018339)) by next generation sequencing (NGS) approach. A library was obtained using a custom panel (NimbleGen SeqCap EZ Technology (Roche)) targeting exons and exon-intron boundaries (+/− 25 bp). Sequencing was performed on a NextSeq500 (Illumina) sequencer. Coverage was 100% at a depth of 30X and the bioinformatic pipeline allowed SNV and CNV detection. The diagnosis of MADD was confirmed by the finding of two new heterozygous *ETFA* substitutions, c.354C > A (p.Asn118Lys) and c.652G > A (p.Val218Met). Sanger sequencing was performed to confirm these pathogenic variants. No other pathogenic genetic variations were detected using an NGS-specific panel.

**Table 1:** Urinary organic acids profile analysis performed by Gas chromatography – mass spectroscopy

	Urinary concentrations in the patient (mmol/mol of creatinine)	Control Range
Adipic acid	1004.2 ↑↑↑	< 4.7
Suberic acid	131.8 ↑↑	< 1.9
Sebacic acid	137.9 ↑	< 9
Glutaric acid	56.0 ↑↑	< 2
Ethylmalonic acid	65.9 ↑	< 8.7
2-hydroxyglutaric acid	33.5 ↑	< 16.4
3-hydroxyglutaric acid	12.5 ↑↑	< 0.4
Hexanoylglycine	20.1 ↑	< 4

Treatment was based on a recommendation of avoiding fasting, a slow-release carbohydrate-rich evening meal, and L-carnitine supplementation (approximately 100 mg/kg/day). This treatment was maintained and associated with riboflavin supplementation (approximately 150 mg/day). This treatment appeared to be sufficient, as the patient exhibited normal development and scholastic performance, with no other metabolic crises during the following months.

## Discussion and conclusion

Multiple acyl-CoA dehydrogenase deficiency exhibits varying clinical symptoms in childhood. This disease has a lower prevalence than that of other diseases involved in the dysfunction of mitochondrial β oxidation, such as MCAD (1/14,600), which was highly suspected in this case before the urinary organic acids profile was obtained. Analysis of the urinary glutaric acid and 2-hydroxyglutaric levels is crucial to distinguish MADD from MCAD. Normal levels direct the diagnosis towards MCAD, whereas mild or high levels direct the diagnosis towards a mild or a severe form of MADD, respectively [4]. Clinical and biological findings observed in a riboflavin metabolism deficit can also mimic those observed in MADD. Indeed, riboflavin is a hydrophilic vitamin involved in flavin adenine dinucleotide (FAD) synthesis, the prosthetic redox group of the heterodimeric ETFA/ETFB, allowing electron transfer. Confirmation of the diagnosis of MADD thus relies on genetic testing of the ETF and ETF-QO encoding genes, as well as those involved in riboflavin metabolism. Various genes can be mutated and are known to promote the reported findings, including those that encode *ETFA, ETFB, ETFDH*, the three cytoplasmic transporters of riboflavin (*SLC52A1*, *SLC52A2*, *SLC52A3*), the mitochondrial transporter of riboflavin (*SLC25A32*), and FAD synthetase *(FLAD1*).

In the present case, we identified substitutions in the *ETFA* gene, whereas sequencing of the other genes mentioned above showed no variations after enrichment by capture of the coding regions. The originality of this work is based on the description of new genetic variations which can contribute to explaining the role of the various amino acids involved in the enzymatic activity of ETFA. According to Grünert et al., only 5% of MADD patients diagnosed after a few years of life carry an *ETFA* variation. The main genes that are generally involved are *ETFDH* (93%) and, to a lesser extent, *ETFB* (2%) [5]. ETF is a heterodimeric protein consisting of two subunits, with no covalent bonds: ETFA (domain I and II) and ETFB (domain III). FAD, which is crucial for ETF activity, is also known to be a cofactor involved in the assembly and stability of the ETF complex, preventing its proteolytic digestion [6]. In addition to FAD, the enzymatic site of ETF is characterized by a direct interaction with adenosine monophosphate (AMP) inside domain III. Several studies have described the critical role of AMP in stabilising the active three-dimensional form of the ETF heterodimer involved in the interaction between the alpha and beta subunits and FAD [7].

The *ETFA* gene is located on chromosome 15q23–25 and contains 12 exons. The variations found in this patient were c.354C > A and c.652G > A, which induce p.Asn118Lys and p.Val218Met substitutions, respectively. The c.354C > A variation (p.Asn118Lys), located at the beginning of exon 5, has never been described in affected patients and is not reported in the GnomAD database. Moreover, according to Alamut Visual analysis, this variation is predicted to be pathogenic by two of the following three software: Mutation Taster and Polyphen2 versus SIFT. The c.652G > A variation (p.Val218Met), located on exon 7, has been detected twice among 251,428 alleles in GnomAD. It is predicted to be ‘probably damaging’ by the three mentioned prediction tools. Genetic investigation of the parents allowed confirmation of a compound heterozygous variation form in this patient, as the father carries the c.354C > A variation and the mother the c.652G > A variation. However, both parents have dominant normal alleles, with no symptomatology. In addition, the CADD phred score for c.354C > A and c.652G > A are 28.1 (pathogenic) and 33 (highly pathogenic), respectively [8]. There is no genetic information concerning the patient’s brother, who is clinically healthy.

**Fig. 1:**
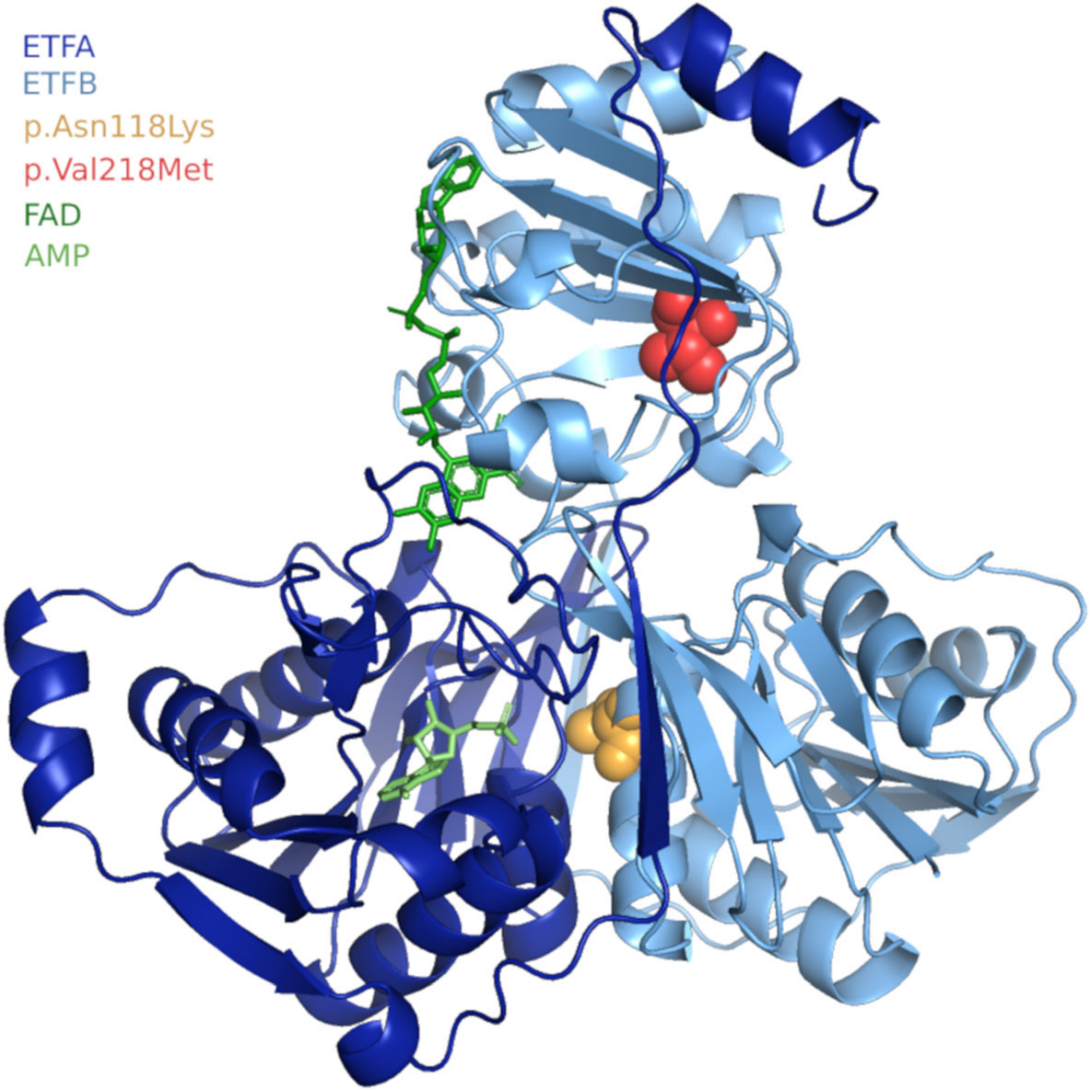
Three-dimensional representation of ETF, a heterodimeric protein composed of two subunits with no covalent bond: ETFA (domain I and II) and ETFB (domain III). The ETFA chain is represented in dark blue, the ETFB chain in light blue, and the ligands AMP in light green and FAD in dark green. The orange ball represents the c.354C > A (p.Asn118Lys) variation and the red ball c.652G > A (p.Val218Met)

We investigated the pathogenicity of these variations using ETFA and ETFB 3D protein models to study the potential effect of the two variations (Fig. 1) [9]. We determined the probable interface between the two subunit proteins using the python interface residues script [10]. The c.354C > A (p.Asn118Lys) variation (orange) is located at the interface between the two subunits and would mainly disturb heterodimerisation. The c.652G > A (p.Val218Met) variation (red) is positioned in a β sheet and is characterized by a valine substituted by a methionine. According to Dynamut software, which predicts the impact of mutations on protein conformation, flexibility, and stability, this variation could destabilize the 3D structure of the ETFA protein and then disturb its redox function [11]. Thus, these two variations would together induce a pathological phenotype. However, the patient did not exhibit MADD symptoms before the episode of decompensation. However, patients with the mild form of MADD often describe muscular weakness, fatigability, nausea, and vomiting because of suboptimal fatty-acid β oxidation in the mitochondria. During the decompensation episode reported here, the patient exhibited seizures resistant to traditional anti-convulsive treatment and partial recovery after intravenous glucose injection. The chromatographic profile of urinary organic acids was contributive and characterized by high levels of glutaric acid, 2-hydroxyglutaric acid, and dicarboxylic acids associated with high levels of short and medium acylcarnitine chains (C4 to C14) in the plasma. Clinical examination was normal, without common signs, such as hepatomegaly, hypotonia, or muscular pain during effort. No muscular or liver biopsies were performed after obtaining the genetic results to avoid such an invasive exam. However, such biopsies are generally only performed to quantify the remaining enzymatic activity or measure the accumulation of adipocytes in the liver, typically found in this disease.

In 2003, Olsen et al. reported a clear relationship between the phenotype and genotype in MADD [12]. Homozygous patients, exhibiting a nonsense mutation with amino acids substitutions near the enzymatic site of ETF, showed neonatal onset of MADD, with a severe phenotype and poor residual enzyme activity (from < 3 to 12% remaining activity). However, if mutations affect a single allele that is not directly involved in the enzymatic activity of ETF, patients exhibit a mild form, with a late onset during childhood or even adulthood. The common symptoms are less severe and are sporadic, characterized mainly by fatigability during episodes of stress. However, the disease can progress towards muscle lesions and respiratory dysfunction, as well as pancreatitis. In the case reported here, the young age of the patient may explain the lack of these types of symptoms.

Riboflavin supplementation is generally proposed for the treatment of MADD, particularly when ETFDH is deficient, associated with the strict avoidance of fasting and high intake of slow-release carbohydrates in the evening. Mild activity and sports are recommended for these patients. D,L-3hydroxybutyrate treatment has also been proposed for riboflavin resistant forms to promote the consumption of ketone bodies [13]. Silmara de Moraes et al. showed that L-carnitine treatment has protective affects against DNA damage in long chain 3-hydroxyacyl-CoA dehydrogenase deficiency, MCAD, and MADD [14]. The authors suggested that L-carnitine could be used as systematic supplementation for these disorders. In addition, El-Gharbawy and Vockley showed that the treatment effect may not be so clear during an episode of MADD. They advised avoiding fasting and medium-chain triglyceride (MCT) oil as supplementation and to combine L-carnitine and glycine intake with a low-fat and protein diet [15]. According to the recent literature, L-carnitine supplementation may provide benefits at several levels. Indeed, it induces a neuroprotective effect in the central nervous system, as described for microglial and endothelial cells of mice with Parkinson’s disease [16]. In addition, carnitine prevents sensory neuron death in peripheral nerve injury and accelerates regeneration [17] and exhibits antioxidant activity in erythrocytes [18]. In our case, the five-year-old patient exhibited normal cognitive development following good compliance and the avoidance of fasting.

This case reports the description of new pathogenic variatnts of the *ETFA* gene, a gene usually associated with severe forms of the disease but associated in this case with a mild form of MADD. The compound heterozygous mutations (p.Asn118Lys and p.Val218Met) in the *ETFA* gene induce the production of moderately altered proteins, leading to a weakly pathogenic form. These findings emphasize the need for further studies on the roles and activity of ETFA.

## Data Availability

The datasets used and/or analysed during the study are available from the corresponding author upon reasonable request.

## Abbreviations

ACTH: Adrenocorticotropic hormone
AMP: Adenosine monophosphate
APGAR: Appearance, pulse, grimace, activity, respiration
ATP: Adenosine triphosphate
ETF: Electron transfer flavoprotein
ETFA: Electron transfer flavoprotein subunit Alpha
ETFB: Electron transfer flavoprotein subunit Beta
ETFDH: Electron transfer flavoprotein Ubiquinone-oxidoreductase
FAD: Flavin adenine dinucleotide
GA II: Glutaric aciduria type II
GCMS: Gas chromatography – mass spectrometry
MADD: Multiple acyl-CoA dehydrogenase deficiency
MCAD: Medium-chain acyl-coenzyme A dehydrogenase deficiency
MCT: Medium-chain triglyceride
NGS: Next generation sequencing
